# The Complete Genome Sequence of the Pathogenic Intestinal Spirochete *Brachyspira pilosicoli* and Comparison with Other *Brachyspira* Genomes

**DOI:** 10.1371/journal.pone.0011455

**Published:** 2010-07-06

**Authors:** Phatthanaphong Wanchanthuek, Matthew I. Bellgard, Tom La, Karon Ryan, Paula Moolhuijzen, Brett Chapman, Michael Black, David Schibeci, Adam Hunter, Roberto Barrero, Nyree D. Phillips, David J. Hampson

**Affiliations:** 1 Centre for Comparative Genomics, Murdoch University, Perth, Western Australia, Australia; 2 Faculty of Informatics, Mahasarakham University, Mahasarakham, Thailand; 3 Animal Research Institute, School of Veterinary and Biomedical Science, Murdoch University, Perth, Western Australia, Australia; University of Hyderabad, India

## Abstract

**Background:**

The anaerobic spirochete *Brachyspira pilosicoli* colonizes the large intestine of various species of birds and mammals, including humans. It causes “intestinal spirochetosis”, a condition characterized by mild colitis, diarrhea and reduced growth. This study aimed to sequence and analyse the bacterial genome to investigate the genetic basis of its specialized ecology and virulence.

**Methodology/Principal Findings:**

The genome of *B. pilosicoli* 95/1000 was sequenced, assembled and compared with that of the pathogenic *Brachyspira hyodysenteriae* and a near-complete sequence of *Brachyspira murdochii*. The *B. pilosicoli* genome was circular, composed of 2,586,443 bp with a 27.9 mol% G+C content, and encoded 2,338 genes. The three *Brachyspira* species shared 1,087 genes and showed evidence of extensive genome rearrangements. Despite minor differences in predicted protein functional groups, the species had many similar features including core metabolic pathways. Genes distinguishing *B. pilosicoli* from *B. hyodysenteriae* included those for a previously undescribed bacteriophage that may be useful for genetic manipulation, for a glycine reductase complex allowing use of glycine whilst protecting from oxidative stress, and for aconitase and related enzymes in the incomplete TCA cycle, allowing glutamate synthesis and function of the cycle during oxidative stress. *B. pilosicoli* had substantially fewer methyl-accepting chemotaxis genes than *B. hyodysenteriae* and hence these species are likely to have different chemotactic responses that may help to explain their different host range and colonization sites. *B. pilosicoli* lacked the gene for a new putative hemolysin identified in *B. hyodysenteriae* WA1. Both *B. pilosicoli* and *B. murdochii* lacked the *rfbBADC* gene cluster found on the *B. hyodysenteriae* plasmid, and hence were predicted to have different lipooligosaccharide structures. Overall, *B. pilosicoli* 95/1000 had a variety of genes potentially contributing to virulence.

**Conclusions/Significance:**

The availability of the complete genome sequence of *B. pilosicoli* 95/1000 will facilitate functional genomics studies aimed at elucidating host-pathogen interactions and virulence.

## Introduction

**Figure 1:**
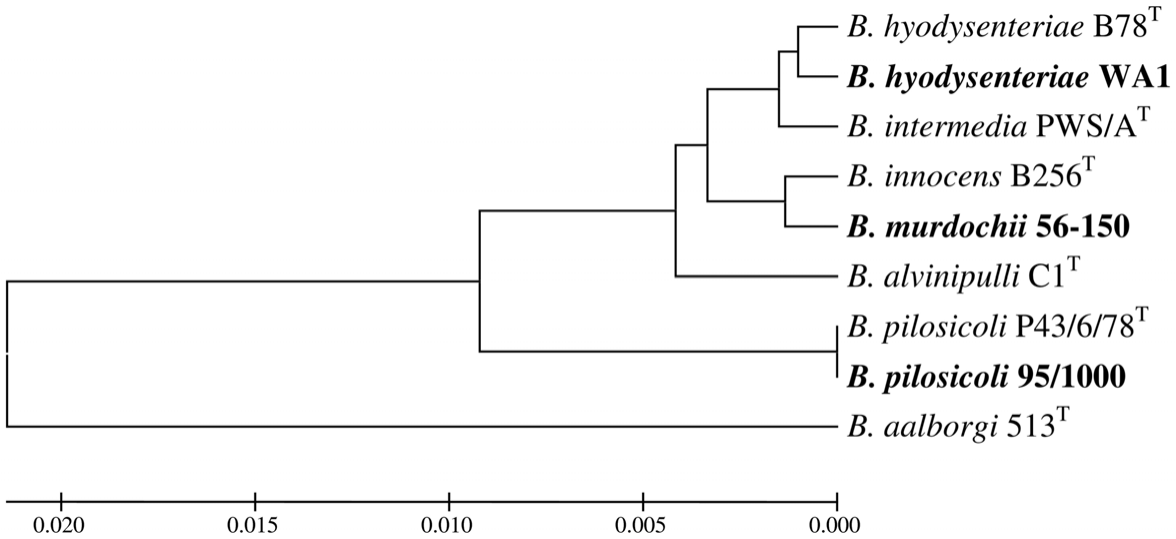
Phylogenetic tree constructed using the UPGMA method showing the relationships of the seven named *Brachyspira* species based on the full sequence of their 16S rRNA genes. The names of the strains that were analysed in this study are marked in bold. 0.002 on the scale bar represents two substitutions in 1000 bp.

Spirochetes of the genus *Brachyspira* are anaerobic bacteria that colonize the large intestine of animals and birds [1]. There are currently seven officially named species in the genus, including both pathogenic and commensal representatives. The names of the species and their phylogenetic relationships based on their 16S rRNA gene sequences are shown in Figure 1. The two main pathogenic species are *Brachyspira hyodysenteriae*, the agent of a major pig disease called swine dysentery, and *Brachyspira pilosicoli*, the cause of a condition known as intestinal (or colonic) spirochetosis. *B. pilosicoli* has a wider host range than *B. hyodysenteriae*, and colonizes a variety of species, including human beings [2]–[6]. Infections with *B. pilosicoli* are particularly common in intensively housed pigs and chickens: colonized individuals may develop focal areas of inflammation in the large intestine, with diarrhea and reduced rates of growth and production. Colonization with *B. pilosicoli* also occurs at a high prevalence rate in human beings living in crowded and unhygienic conditions, particularly in developing countries [7]–[11], as well as amongst homosexual males [12]. Despite the potential importance of *B. pilosicoli* as a pathogen, it has not been extensively studied. Progress has been hampered by the spirochete's specialised growth requirements and slow growth rate, as well as by a lack of genomic information and an absence of means for genetic manipulation.

A characteristic feature of infection with *B. pilosicoli* is the attachment of the spirochete by one cell end to the luminal surface of enterocytes in the large intestine; with time, dense mats of these attached bacteria may form a “false brush-border” covering the whole luminal surface of the enterocytes [12], [13]. Experimentally, following attachment to cultured Caco-2 cells *B. pilosicoli* has been shown to induce apoptosis, actin rearrangement and increased expression of interleukin-1ß (IL-1ß) and IL-8 [14]. Such pathological changes may contribute to the local colitis and diarrhea that is observed *in vivo*.

The recent publication of the genome sequence of *B. hyodysenteriae* strain WA1 [15] and the availability of a near-complete genome sequence of *Brachyspira murdochii* type strain 56-150^T^ (DSM 12563) in GenBank represent important opportunities to advance research on these *Brachyspira* species. *B. murdochii* is generally considered to be a harmless commensal in pigs, chickens and rats [16], [17]; however, there have been recent reports that it may have some potential to cause colitis in pigs [18], [19]. In the current study the genome of *B. pilosicoli* strain 95/1000 was sequenced and subjected to comparative genomic analysis, particularly in relation to the sequences of WA1 and 56-150^T^. As the sequence of the latter was incomplete some comparisons were not appropriate. The overall aim of this work was to enhance knowledge about *B. pilosicoli* and its relationships to the other *Brachyspira* species, particularly in regard to identifying the genetic basis of their different ecologies and pathogenic potentials.

## Materials and Methods

### Spirochete strain and growth conditions

*Brachyspira pilosicoli* strain 95/1000 (ATCC BAA-1826) was originally isolated in Western Australia from the diarrheic feces of a commercial pig with porcine intestinal spirochetosis [13]. The spirochete was purified by repeated subculture, grown to mid-log phase in pre-reduced anaerobic broth [20], and a cell pellet prepared.

### Genomic DNA preparation, library construction and sequencing

Preparation of genomic DNA, library construction and sequencing was as previously described for *B. hyodysenteriae* [15]. Sequencing was undertaken at the Australian Genome Research Facility. The first round of sequencing was performed via Sanger sequencing, with a total of 42,565 reads generated. The second round was performed using a pyrosequencing approach on a Roche-454 GS20 instrument, generating more than 25 times coverage of the genome. The quality filtered reads were assembled into contiguous sequences using the Newbler Assembler software (http://www.454.com/). To finish the genome sequence, remaining gaps were closed by PCR walking between un-linked contiguous sequences [21].

### Sequence analysis and annotation

For both the newly sequenced *B. pilosicoli* 95/1000 and for the incomplete genome sequence of *B. murdochii* 56-150^T^ obtained from GenBank (ABTG00000000), sequence analysis and annotation were as previously described for *B. hyodysenteriae* WA1 [15]. Dot matrix plots comparing the genomes of 95/1000, 56-150^T^ and WA1 were generated using Freckle, an in-house development of the Dotter tool [22]. The minimum size of matched sequences was set to 20 base pairs (bp).

The complete nucleotide sequence and annotation of *B. pilosicoli* 95/1000 has been deposited in GenBank (accession number CP002025; Project ID: 48097). Annotations and functional assignments for *B. pilosicoli* 95/1000 and for *B. murdochii* 56-150^T^ also can be accessed at the CCG website (http://ccg.murdoch.edu.au/spirochaetales/).

## Results and Discussion

### General genome features

**Figure 2:**
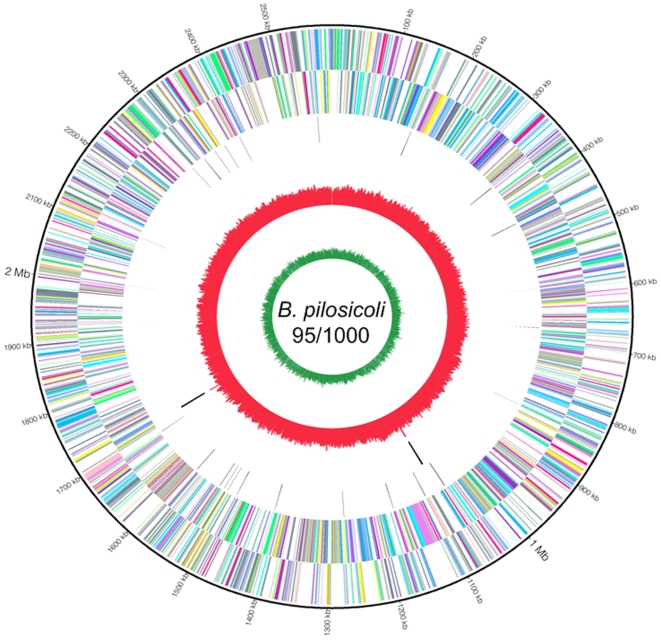
Genome of *B. pilosicoli* 95/1000. Circles outer to inner: Genes, forward strand, COG coded; Genes, reverse strand, COG coded; tRNA; rRNA; GC skew; AT skew. All genes are color-coded according to Cluster of Orthologous Gene Categories (COG) functions: violet for translation, ribosomal structure and biogenesis; plum for RNA processing and modification; pink for transcription; deep pink for DNA replication, recombination and repair; hot pink for chromatin structure and dynamics; wheat for cell division and chromosome partitioning; light salmon for nuclear structure; yellow for defence mechanisms; gold for signal transduction mechanisms; pale green for cell envelope biogenesis, outer membrane; spring green for cell motility and secretion; lawn green for cytoskeleton; yellow green for extracellular structures; aquamarine for intracellular trafficking, secretion, and vesicular transport; medium aquamarine for posttranslational modification, protein turnover, chaperones; cyan for energy production and conversion; deep sky blue for carbohydrate transport and metabolism; sky blue for amino acid transport and metabolism; light slate blue for nucleotide transport and metabolism; orchid for coenzyme metabolism; medium orchid for lipid metabolism; dark orchid for inorganic ion transport and metabolism; blue violet for secondary metabolites biosynthesis, transport and catabolism; slate grey for general function prediction only; grey for function unknown; grey for not in COGs; black for tRNA.

The general features of the genome of *B. pilosicoli* 95/1000, together with those of *B. hyodysenteriae* WA1 and the near-complete genome of *B. murdochii* 56-150^T^ are summarised in Table 1. The complete genome of *B. pilosicoli* 95/1000 consisted of a single circular chromosome of 2,586,443 bp (Figure 2), making it the smallest of the three *Brachyspira* genomes analysed to date (with *B. murdochii* being the largest). The overall G+C content of the *B. pilosicoli* genome was 27.9 mol%. Unlike *B. hyodysenteriae* WA1, *B. pilosicoli* 95/1000 did not contain a ∼36 Kb plasmid. Based on the available sequence it also appeared unlikely that *B. murdochii* contained a similar plasmid. The *B. pilosicoli* genome encoded 2,338 genes, with an overall 85% coding region. In comparison, *B. murdochii* 56-150^T^ had 3,055 predicted genes in the seven contigs that were available. The percent coding region was similar across the three genomes. The average size of the predicted genes in *B. pilosicoli* 95/1000 was 997 bp, and tentative function was assigned to 1,645 of these. The *B. pilosicoli* strain had 569 more functionally assigned genes than did *B. murdochii* 56-150^T^ and 282 less than *B. hyodysenteriae*. *B. pilosicoli* 95/1000 had 655 genes with unknown function and this was somewhat fewer than for *B. hyodysenteriae* (704), but much fewer than the extremely high number of unmatched genes for *B. murdochii* (1,925). A total of 1,201 (51.4%) genes in *B. pilosicoli* had matches in the COG database and 1,410 (60.3%) had matches in the KEGG database (Table 1). Of these, only 426 (18.2%) were assigned to enzymes (EC number) and 635 (27.2%) were involved in KEGG pathways. Of the *B. pilosicoli* open reading frames (ORFs) screened, 244 were predicted to encode proteins with signal peptides and 48 were predicted to have transmembrane helices. Eleven genes encoded proteins predicted to have seven transmembrane helices.

**Table 1:** General genomic features predicted for *B. pilosicoli* 95/1000 and comparison with those of *B. hyodysenteriae* WA1 and *B. murdochii* 56-150^T^.

Feature	*B. pilosicoli* 95/1000	*B. hyodysenteriae* WA1	*B. murdochii* 56-150^T^ *
Genome size (bp)	2,586,443	3,036,634	3,189,383
Coding region (%)	85%	86%	86%
G+C content	27.90%	27.06%	26.91%
Total genes	2,338	2,669	3,055
rRNA genes	3	3	3
tRNA genes	35	35	36
Hypothetical protein/Conserved hypothetical protein	655	704	1,925
Genes with function prediction	1,645	1,927	1,076
Protein–coding genes	1,987	2,153	2,997
Number of multiple copies of gene (%)	99 (4.3)	210 (7.9)	204 (6.7)
Genes assigned to COG	1,201	1,217	1,217
Genes assigned to KEGG	1,410	1,492	1,509
Genes assigned E.C. numbers	426	509	436
Genes with signal peptide	244	360	220
Genes with transmenbrane helices	48	52	34

*incomplete genome containing 7 contigs (GenBank accession number ABTG00000000).

The three *Brachyspira* species had similar numbers of tRNA genes, representing all 20 amino acids (Table 1). As with *B. hyodysenteriae* [15], only single copies of the 16S, 23S, and 5S ribosomal RNA genes were found in *B. pilosicoli* and *B. murdochii*. In *B. pilosicoli* 95/1000, the *rrs* and *rrl* genes were closely linked, with the *rrl* (16S) gene being about 645 Kb from the other two genes. This rRNA gene organisation has been noted earlier; it distinguishes the *Brachyspira* species from other spirochetes, and presumably pre-dates speciation in the *Brachyspira* genus [23]. Although spirochetes have a monophyletic origin, the copy number and organisation of rRNA genes differ in the different genera. For instance, the *Treponema pallidum* rRNA genes appear to be arranged in two typical *rrn* operons [24], [25]. A single rRNA locus is found in most *Borrelia* species, with *rrs* separated from *rrl* and *rrf* by a small segment of DNA (∼4 Kb). In *Borrelia burgdorferi* the *rrf-rrl* cluster is duplicated and is found immediately adjacent to the *rrs-rrl-rrf* cluster [26]. Pathogenic *Leptospira* species possess two copies each of *rrs* and *rrl* and one copy of *rrf* [27]. The non-pathogenic *Leptospira biflexa* contains two copies of each rRNA gene [24], and these are dispersed around the genome. Differences in the sequences of the 16S rRNA genes have been used to examine phylogenetic relationships between spirochete genera and species [28]. On this basis, in the case of the *Brachyspira* species, *B. pilosicoli* is more distantly related to *B. hyodysenteriae* than is *B. murdochii* (Figure 1).

**Figure 3:**
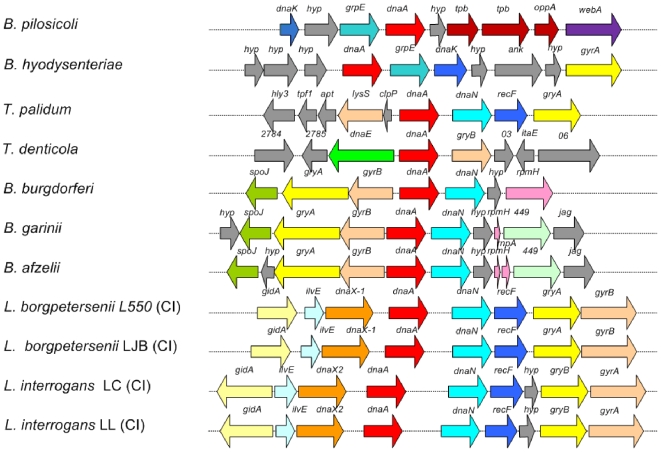
Comparison of the *B. pilosicoli* and *B. hyodysenteriae* putative origins of replication (oriC) region with that of other spirochetes. Homologous genes commonly found at bacterial origins are indicated in similar colors. *dnaA* is indicated in red and hypothetical proteins are indicated in grey. Conservation of a cluster of genes is located around the origin of replication in several spirochetes. The genes encoding the putative proteins and the origins of replication are indicated.

The origin and terminus of replication in the genome of *B. pilosicoli* 95/1000 were predicted based on the position of *dnaA* (BP951000_0595), as bacterial chromosome replication origins are typically located near this gene. The putative *oriC* origin of replication was identified in an AT-rich intergenic region upstream of *dnaA*, in the vicinity of a cluster of hypothetical DnaA boxes. Similar putative *oriC*s with comparable DnaA boxes have been identified in other spirochete genomes (Figure 3). The original of replication in *B. murdochii* was not found due to the genome sequence being incomplete. The putative origins of replication are located centrally within the most highly conserved and synteneous regions of the various spirochete genomes (Figure 3). *B. pilosicoli* contained the genes *grpE*, *dnaK*, *hyp*, *ark*, *hyp*, *arg* and *gyrA* downstream of the *dnaA*, an arrangement similar to *B. hyodysenteriae* WA1 [15]. *B. pilosicoli* had a unique hypothetical coding region immediately upstream of the *dnaA* gene, whereas there were three hypothetical protein encoding genes upstream of *dnaA* in *B. hyodysenteriae*. This finding suggests that in *B. pilosicoli* 95/1000 and *B. hyodysenteriae* WA1 *oriC* relocated during evolution, presumably as the result of a DNA rearrangement. The differences in the origins of replication compared to other spirochetes suggest that there could be different mechanisms for replication of the spirochete chromosomes. Experimental studies will be required to verify the origin of replication in *B. pilosicoli*, as has been accomplished for *Borrelia burgdorferi* [29].

### Whole-genome alignment

**Figure 4:**
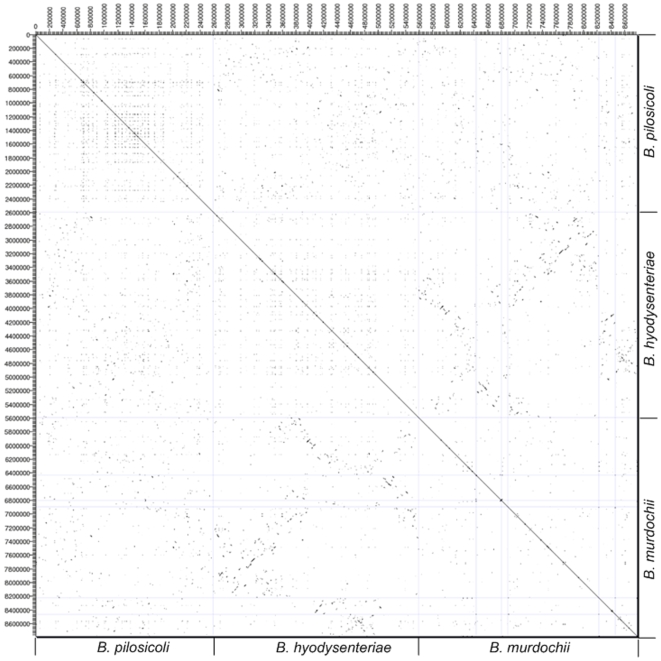
Dot matrix plots comparing the genomes of 95/1000, 56-150^T^ and WA1 generated using Freckle. The output displays a two dimensional plot with dots representing matched regions between the three genomes. The minimum size of matched sequences was set to 20 bp.

A dot plot comparison of the genomes of *B. pilosicoli*, *B. hyodysenteriae* and *B. murdochii* showed no large scale conservation of gene order, but only conservation of some genes in clusters (Figure 4). Gene cluster conservation appeared to be greatest between *B. hyodysenteriae* and *B. murdochii*, consistent with these two species being phylogenetically more closely related to each other than to *B. pilosicoli*. A 26 Kb region of unknown significance that is partially conserved in *B. hyodysenteriae* WA1, *B. pilosicoli* 95/1000, *Enterococcus faecalis* and *Escherichia coli* [30], also was identified in *B. murdochii*. Alignment of the two complete *Brachyspira* genomes using the Artemis Comparison Tool [31] identified extensive gene rearrangements between them (Figure S1 in supporting information), consistent with the findings of an earlier comparison of partial physical maps of the two species [32].

**Figure 5:**
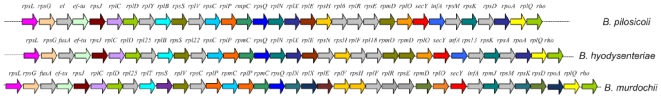
Conservation of the gene cluster for ribosomal proteins in *B. pilosicoli* 95/1000, *B. hyodysenteriae* WA1 and *B. murdochii* 56-150^T^.

An exception to the apparent general lack of conservation of gene order between the three *Brachyspira* genomes was a high conservation of the operons encoding ribosomal proteins. *B. pilosicoli* 95/1000 contained genes for 33 *r*-proteins organised in a 18 Kb region, whereas the ribosome clusters in the *B. hyodysenteriae* and *B. murdochii* strains included a total of 32 and 36 genes encoding *r*-proteins organised in 15 Kb and 18 Kb regions, respectively (Figure 5). The sequences of the *B. pilosicoli r*-proteins in this cluster were highly similar to the *B. hyodysenteriae* and *B. murdochii* homologs. Ribosomal protein genes are interesting because of their ubiquity and similar conservation rate such that horizontal transfer between lineages is unlikely. Apparently there has been a positive selection for clustering of these physically interacting proteins in the sequenced *Brachyspira* species, whilst there has been no absolute requirement for juxtaposition of other genes in the genomes. Synteny therefore has been lost at a much faster rate than is useful for prediction of gene function. This is consistent with the situation in other bacterial species, where orthologous genes are not necessarily located at the same relative position in the genomes, and only certain gene clusters are syntenic [33]. Genomes of closely related species typically maintain a high degree of synteny [34], whereas genomes of moderately distant species, such as these *Brachyspira* species, usually have no significant overall synteny [35].

### Functional predictions amongst the *Brachyspira* species

The COG categories of the protein-coding genes are shown in Table 2. Generally there were few significant differences amongst the three genomes, although the smaller genome of *B. pilosicoli* 95/1000 contained more genes than the other two species in five categories: (N) Cell motility, (U) Intracellular trafficking, secretion, and vesicular transport, (C) Energy production and conversion, (H) Co-enzyme transport and metabolism, and (I) Lipid transport and metabolism. On the other hand, *B. hyodysenteriae* had more genes functioning in (K) Transcription, (V) Defense mechanisms, (T) Signal transduction mechanisms, (O) Posttranslational modification, protein turnover, chaperones, (G) Carbohydrate transport and metabolism, (E) Amino acid transport and metabolism, (P) Inorganic ion transport and metabolism, and (Q) Secondary metabolites biosynthesis, transport and catabolism. *B. murdochii* had more genes involved with (L) Replication, recombination and repair, and (M) Cell wall/membrane/envelope biogenesis. It was noteworthy that *B. pilosicoli* had the largest number of genes involved in energy production and conversion, and this capacity may enhance its potential to colonize various hosts.

**Table 2:** Distribution of Cluster of Orthologous Genes (COGs) in *B. pilosicoli* 95/1000, *B. hyodysenteriae* WA1 and *B. murdochii* 56-150^T^, showing the number and percentage of proteins within a genome assigned to each functional group (the *B. murdochii* genome is incomplete).

Functions		*B. pil*	%	*B. hyo*	%	*B. mur*	%
Cellular Processes							
J	Translation, ribosomal structure and biogenesis	122	5.30	122	4.57	120	4.00
K	Transcription	51	2.21	63	2.36	46	1.53
L	Replication, recombination and repair	51	2.21	51	1.91	63	2.10
Cellular Processes and Signalling							
D	Cell cycle control, cell division, chromosome partitioning	10	0.43	10	0.37	9	0.30
V	Defence mechanisms	35	1.52	40	1.50	32	1.07
T	Signal transduction mechanisms	16	0.69	21	0.79	18	0.60
M	Cell wall/membrane/envelope biogenesis	74	3.21	75	2.81	81	2.70
N	Cell motility	40	1.74	38	1.42	38	1.27
U	Intracellular trafficking, secretion, and vesicular transport	11	0.48	10	0.37	8	0.27
O	Posttranslational modification, protein turnover, chaperones	40	1.74	41	1.54	37	1.23
Metabolism							
C	Energy production and conversion	89	3.86	83	3.11	82	2.74
G	Carbohydrate transport and metabolism	101	4.39	108	4.05	104	3.47
E	Amino acid transport and metabolism	141	6.12	148	5.55	115	3.84
F	Nucleotide transport and metabolism	49	2.13	48	1.80	49	1.63
H	Coenzyme transport and metabolism	47	2.04	42	1.57	46	1.53
I	Lipid transport and metabolism	41	1.78	37	1.39	40	1.33
P	Inorganic ion transport and metabolism	53	2.30	74	2.77	73	2.44
Q	Secondary metabolites biosynthesis, transport and catabolism	9	0.39	16	0.60	10	0.33
Poorly characterised							
R	General function prediction only	149	6.47	171	6.41	175	5.84
S	Function unknown	72	3.13	70	2.62	71	2.37
Unassigned							
X	not in COG	1,201	52.15	1,401	52.49	1,780	59.39
	Total	2,303	100	2,669	100	2,997	100

Previously it has been shown that *B. hyodysenteriae* lacks many genes for the biosynthesis of small molecules, and therefore must acquire these from the environment [15]. Interestingly, *B. pilosicoli* was predicted to contain substantially fewer genes involved in inorganic ion transport and metabolism (P) than both *B. hyodysenteriae* and *B. murdochii* (53 versus 74 and 73, respectively; Table 2). Furthermore, it is not known which *B. pilosicoli* genes may act to compensate for the shortage of such biosynthetic pathways, since the substrates of many of the genes regarded as encoding transporters due to their possession of the motif sequences were unknown. These differences were unexpected, given that *B. pilosicoli* has a wider host range than the other two *Brachyspira* species, and hence might be predicted to have a greater metabolic capacity and flexibility in order to survive in these more varied nutritional environments.

### Global gene comparisons between the *Brachyspira* species

**Figure 6:**
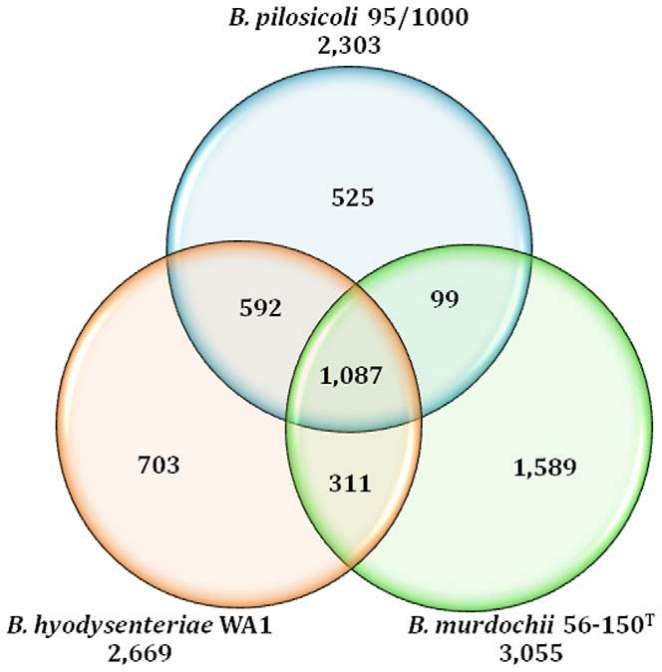
Global gene conservation in the three *Brachyspira* species strains. Each circle represents the total number of gene types in each genome. Overlapping regions depict the number of genes types shared between the respective genomes. The numbers outside the circles indicate the total number of CDS identified in each genome.

As expected, comparative analysis of the *B. pilosicoli* genome across available microbial genomes in the non-redundant (*nr*) database at NCBI identified greatest similarities with *B. hyodysenteriae* and *B. murdochii*. However, as with *B. hyodysenteriae* [15], the next highest levels of similarities at the protein level were with *Clostridium* species (∼10%) and *E. coli* (∼5%). The three sequenced *Brachyspira* species contained a total of 1,087 conserved or “core” genes (Figure 6). It would be instructive to determine whether these same genes are conserved in other *Brachyspira* species. The majority of genes in *B. pilosicoli* (1,769, 77%) were conserved in *B. hyodysenteriae*; therefore, most of the genome-inferred metabolic potential of *B. hyodysenteriae* described previously can be extrapolated to *B. pilosicoli*. Only an additional 99 genes were conserved between *B. pilosicoli* and *B. murdochii*, whereas an additional 311 were conserved between *B. hyodysenteriae* and *B. murdochii*. *B. hyodysenteriae* had 1,014 genes that were not shared with *B. pilosicoli*, whereas *B. murdochii* contained a remarkable 1,900 genes not found in *B. pilosicoli*. On this basis, *B. pilosicoli* seemed to be more similar to *B. hyodysenteriae* than to *B. murdochii*. A large proportion of the unique genes in both *B. hyodysenteriae* and *B. murdochii* were of unknown function (Table 1).

Although the *B. hyodysenteriae* genome was ∼450 Kb larger than that of *B. pilosicoli*, both species contained roughly the same number of gene across the identified functional gene categories, and only a relatively small number of genes in *B. pilosicoli* were obviously different from those in *B. hyodysenteriae*. Of the 525 potential genes that were specific for *B. pilosicoli*, 319 were of unknown function. The remaining 206 functionally annotated *B. pilosicoli*-specific genes included those predicted to be involved in energy production, carbohydrate metabolism, amino acid metabolism, capsule biosynthesis (although *B. pilosicoli* is not known to have a capsule), or encoding transcriptional regulators, transporters and predicted surface antigens, as well as forming a bacteriophage region. Selected differences found in *B. pilosicoli* that appear to be potentially significant are described below.

#### Bacteriophage genes

**Figure 7:**
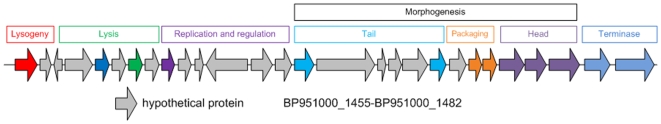
Organisation of the bacteriophage genome in *B. pilosicoli* 95/1000.

*Brachyspira pilosicoli* 95/1000 contained a 25 Kb region (location 1,595,515–1,615,675 bp) encoding components of an integrated bacteriophage (Figure 7). The 29 associated genes in the region were absent from *B. hyodysenteriae* and *B. murdochii*, apart from the OrfG VSH-1 protein homolog that has been associated with a gene transfer agent in *B. hyodysenteriae*. Sixteen genes were identified as encoding hypothetical proteins, whilst 13 were bacteriophage-associated: putative integrase XerDC family protein (BP951000_1455); OrfG VSH-1 phage protein homolog (BP951000_1459); putative Lys - endolysin; glycoside hydrolase (BP951000_1461); putative RNA polymerase domain-containing protein (BP951000_1463); phage tail tape measure protein homolog, TP901 family (BP951000_1469); bacteriophage TP901-1 like family protein homolog (BP951000_1474); putative DNA packaging, Phage QLRG family protein (BP951000_1476); phage major capsid protein homolog, HK97 family (BP951000_1477); phage prohead protease (BP951000_1478); putative phage portal protein, HK97 family (BP951000_1479); DNA methylase N-4/N-6 domain protein homolog (BP951000_1480); putative phage terminase, small subunit, P27 family protein (BP951000_1481); and putative phage terminase, large subunit (BP951000_1482). The GC content in this bacteriophage region was 29.65%, which was slightly higher than the average for the whole genome (27.90%). It is not yet clear whether this integrated bacteriophage is functional, or capable of transferring genetic material between *B. pilosicoli* strains. If it is, it could be developed into a tool for genetic manipulation of the spirochete. By analogy with other prophages, it potentially also could be involved in lysogenic conversion, for example modifying the spirochete phenotype to enhance its virulence [36]. It will be important to determine how widespread and conserved this bacteriophage region is amongst *B. pilosicoli* strains. *B. hyodysenteriae* WA1 also contained two genes predicted to be bacteriophage-associated: phage terminase large subunit (*xtmB*, BHWA1_01969) and integrase (BHWA1_02688), but these were distinct from the bacteriophage genes found in *B. pilosicoli*. A predicted integrase-recombinase protein (4083292.C14.orf00918) was found in *B. murdochii*, but it only had 25.61% identity at the protein level.

The novel bacteriophage region in *B. pilosicoli* 95/1000 was distinct from the smaller prophage-like gene transfer agent (GTA) that previously was described in the genomes of *B. pilosicoli* 95/1000 and in *B. hyodysenteriae* WA-1 [37], and which was originally described in *B. hyodysenteriae* B204 as VSH-1 [38], [39]. The genes of the GTAs in 95/1000 and WA1 were contiguous, but showed gene rearrangements. Elements of the GTA also have been detected in different contigs of a partial genome sequence of *B. intermedia* HB60 [37]. In the current analysis OrfE was found immediately in front of the other 17 GTA genes that were previously described in *B. pilosicoli* 95/1000 [37]. *B. murdochii* 56-150^T^ was found to have a cluster of 31 genes on contig 4083292.C21 (orf00108–orf00140) that included many of the described components of the GTA, as well as 17 hypothetical proteins. Genes for Hvp19/Hvp22, Hvp13, Hvp38, and Hvp28 were not identified in the partial genome of *B. murdochii*. Again, the known genes showed rearrangements from the order found in *B. hyodysenteriae* and *B. pilosicoli*, and it is unclear whether the *B. murdochii* GTA is functional.

#### Genes of the glycine reductase complex system

**Figure 8:**
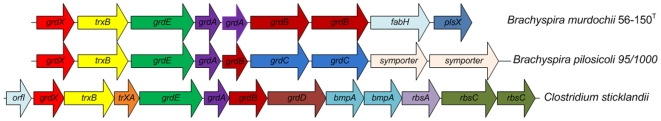
Physical map of the glycine reductase genes and adjacent regions in *B. pilosicoli* 95/1000, *B. murdochii* 56-150^T^ and *Clostridium sticklandii*.

*B. pilosicoli* 95/1000 and *B. murdochii* 56-150^T^ differed from *B. hyodysenteriae* WA1 in that they possessed nine genes within a glycine reductase complex (*grd* cluster): this arrangement is similar to that in the anaerobic bacterium *Clostridium sticklandii* [40] (Figure 8). The locus in *B. pilosicoli* included genes encoding the GrdX protein (*grdX*, BP951000_1852), thioredoxin reductase (*trxB*, BP951000_1853), glycine reductase (*grdE*; BP951000_1854), sarcosine reductase (*grdA*, BP951000_1855), glycine reductase complex selenoprotein B (*grdB*, BP951000_1856), two copies for the glycine/sarcosine/betaine reductase complex, component C, alpha subunit (*grdC*, BP951000_1857 and BP951000_1858), and two copies for a sodium∶alanine symporter family protein (BP951000_1859 and BP951000_1860). In *B.murdochii*, the locus consisted of genes encoding putative glycine reductase complex component (*grdX*, 4083292.C42.orf00552) thioredoxin reductase (*trxB*, 4083292.C42.orf00553), glycine/sarcosine/betaine reductase component B alpha/beta subunit (*grdE*; 4083292.C42.orf00554), two copies of glycine reductase complex selenoprotein A (*grdA*, 4083292.C42.orf00556 and 4083292.C42.orf00557), two copies of selenoprotein B, glycine/betaine/sarcosine/D-proline reductase family (*grdB*, 4083292.C42.orf00558 and 4083292.C42.orf00559), 3-oxoacyl-(acyl-carrier-protein) synthase III (*fabH*, 4083292.C42.orf00560), and fatty acid/phospholipid biosynthesis enzyme (*plsX*, 4083292.C42.orf00561).

ORF *grdX* preceded the thioredoxin/glycine reductase gene cluster in both *B. pilosicoli* and *B. murdochii*, and it is known to be co-transcribed in *C. sticklandii* [40]. An additional two copies of *trxB* were identified in the *B. pilosicoli* genome (BP951000_1651 and BP951000_1853). Other genes in *B. pilosicoli* and *B. murdochii* that were outside the cluster and which encoded proteins with predicted selenocysteine sites were thioredoxin (BP951000_0519 and BP951000_1639; 4083292.C42.orf00619 and 4083292.C42.orf00482) and glutathione peroxidase (BP951000_2065 and BP951000_2066; 4083292.C16.orf00268). Given the potential role of these selenoproteins in antioxidant functions, deficiency of selenium may have detrimental effects on their ability to withstand oxidative stress.

The glycine reductase complex catalyses the reductive deamination of glycine to acetylphosphate and ammonia with the generation of ATP from ADP and orthophosphate [41]. *B. pilosicoli* and *B. murdochii* therefore were predicted to be able to utilise glycine anaerobically as a sole source of carbon and energy, and hence have a distinct energy-conserving mechanism using an internal reaction in which glycine serves as an electron donor during oxidation by a glycine cleavage system, or as an electron acceptor when being reduced by glycine reductase. In contrast, the lack of this complex in *B. hyodysenteriae* suggested that it is unable to ferment glycine to act as a carbon and energy source. The difference between the species suggests that high levels of glycine might favor *B. pilosicoli* and *B. murdochii* populations over *B. hyodysenteriae*, either *in vitro* or *in vivo*. The glycine reductase complex is predicted to play an important role in allowing *B. pilosicoli* to successful colonize a broader range of host species than *B. hyodysenteriae*, and it also may confer advantages to *B. murdochii*. Clearly, further research is required to determine whether glycine does enhance growth of *B. pilosicoli* and *B. murdochii*, as predicted.

The absence of the *grd* cluster in *B. hyodysenteriae* may make it unable to synthesise glutathione and glutaredoxins, and therefore it was predicted to be less able to withstand oxidative stress than the other two species. Only two copies of a gene encoding thioredoxin reductase were identified in *B. hyodysenteriae* (*trxB*, BHWA1_02087 and BHWA1_00602). The predicted ability of *B. pilosicoli* to rapidly respond to an oxidative stress or a redox insult was consistent with its reported ability to survive outside the host for a longer period than *B. hyodysenteriae* [42], [43], and also may explain why it is able survive in more oxygenated host tissues than *B. hyodysenteriae*, such as in the bloodstream of immunocompromised patients [44]. *B. murdochii* also has been isolated from extra-intestinal sites [45]. This predicted ability to withstand oxidative stress also may make *B. pilosicoli* more adaptable in terms of its abilities to colonize the large intestines of a wide variety of host species, where environmental conditions are likely to vary.

#### Sulfatase genes

Three copies of genes encoding sulfatases (BP951000_0858, BP951000_0859 and BP951000_0861) were identified in *B. pilosicoli* 95/1000, whilst one putative copy was found in *B. murdochii* (4083292.C42.orf00884), and none in *B. hyodysenteriae*. Sulfatases play important roles in the cycling of sulfur in the environment, in the degradation of sulfated glycosaminoglycans and glycolipids in the lysosome, and in remodeling sulfated glycosaminoglycans in the extracellular space. The sulfatase genes potentially could encode for enzymes that modify gycosaminoglycans to generate binding sites required for attachment of *B. pilosicoli* [46].

#### Fucosyltransferase genes

A novel set of two copies of genes encoding alpha-1, 2-fucosyltransferase (*fucT*, BP951000_1232 and BP951000_1235) and beta-1, 3-galactosyltransferase (BP951000_1768) were identified in *B. pilosicoli* 95/1000: these are key enzymes in the biosynthesis of Lewis antigens, structures found on the surface of human erythrocytes and epithelial cells. The gastric pathogen *Helicobacter pylori* can express Lewis and related antigens in the O-chains of its surface lipopolysaccharide [47], and this activity is believed to be important for bacterial persistence, immune evasion, and possibly generation of inflammation [48]. Further work is required to determine whether *B. pilosicoli* expresses Lewis antigens, and to investigate their potential involvement in the pathogenesis of infection by this spirochete species.

#### Sialidase genes

Three copies of a gene encoding a sialidase (neuraminidase) family protein homolog (*nanA*, BP951000_2021, BP951000_2022 and BP951000_2023) were identified in *B. pilosicoli* 95/1000, but not in *B. hyodysenteriae* or *B. murdochii*. NanA proteins are produced by a wide variety of mucosal pathogens, and are a potential virulence factor in bacteria [49], [50]. As they are widespread and conserved among a very broad range of important human pathogens, this implies that they have a critical role in microbial ecology [51]. These enzymes may enhance *B. pilosicoli* colonization or induce tissue damage.

#### Aconitase gene

*B. pilosicoli* and *B. murdochii* were both found to have a gene encoding aconitase (BP951000_0370 and 4083292.C42.orf00530, respectively), and this was not present in *B. hyodysenteriae*. Aconitase is a tricarboxylic acid (TCA) cycle protein that catalyses the conversion of citrate to isocitrate, and, amongst other things, has been implicated in the virulence of *Staphylococcus aureus* [52]. It is possible that the aconitase gene may contribute to the control of virulence factor synthesis [53]. As none of the three anaerobic *Brachyspira* species contained genes for a complete TCA cycle, this suggests that retention of the aconitase gene may have an important functional significance. Two other genes were located adjacent to the aconitase gene in both *B. pilosicoli* and *B. murdochii*, these being putative citrate synthase (BP951000_0368 and 4083292.C42.orf00523) and putative isocitrate dehydrogenase (BP951000_0369 and 4083292.C42.orf00525). They were not identified in *B. hyodysenteriae* WA1. The presence of aconitase implies that *B. pilosicoli* and *B murdochii* can utilise the TCA to produce glutamate, and this capacity is discussed later in the section on central metabolic pathways.

### Potential virulence factor screening

Independent of the previous analysis, screening of all coding sequences (CDS) of the three species using Blast, SignalP, PSORT and TMHMM to look for similarities to genes reported to be associated with virulence in other bacteria identified a total of 235 genes with putative roles in virulence in *B. pilosicoli* 95/1000, compared to 303 in *B. hyodysenteriae* WA1 and 142 in *B. murdochii* 56-150^T^. The number of genes in the different categories for all three *Brachyspira* species is shown in Table 3. Interpretation of the results for *B. murdochii* was complicated by the fact that the genome was incomplete, and additional genes are likely to be identified once a complete genome sequence becomes available. Overall, apart from what is shown, the predicted gene products did not have significant similarities with those of other well-characterized toxins or adhesins described in the major species of enteropathogenic bacteria - such as in *E. coli* or *Clostridium* species. Nevertheless, it is important to remember that other virulence-associated genes are likely to exist amongst those that are currently recorded as encoding proteins that are hypothetical or of unknown function.

**Table 3:** Genes with potential roles in pathogenesis and virulence in *B. pilosicoli* 95/1000, and comparison with those in *B. hyodysenteriae* WA1 and *B. murdochii* 56-150^T^.

Putative gene	*B. pil*	*B. hyo*	*B. murd* *
Core genes involved in lipopolysaccharide biosynthesis**	25	30	10
Chemotaxis			
Putative methyl-accepting chemotaxis protein	6	10	7
methyl-accepting chemotaxis protein A (*mcpA*)	2	8	8
methyl-accepting chemotaxis protein B (*mcpB*)	6	19	16
methyl-accepting chemotaxis protein C (*mcpC*)	-	3	1
chemotaxis protein	13	12	7
Flagella	32	33	33
Adhesion and/or surface protein			
Lipoprotein	13	34	6
Variable surface protein	3	9	6
Outer membrane protein	6	4	2
Integral membrane protein	1	6	-
Inner membrane protein	2	7	-
Host cell membrane degradation			
Hemolysis	8	8	4
Phospholipase	2	4	3
Peptidase	39	40	21
Protease	15	15	14
Oxidative stress (*nox*)	2	2	2
Phage	29	2	-
Ankyrin-like protein	31	57	2
Total	235	303	142

*Incomplete genome.

**core LOS biosynthesis genes.

It was of interest that *B. hyodysenteriae*, the most pathogenic of the three species, contained more genes involved with lipopolysaccharide biosynthesis, motility and chemotaxis, and adhesion and/or surface proteins than did the other two species. The latter two did not have more of any category of these potential virulence-associated genes, apart from the bacteriophage genes in *B. pilosicoli*.

#### Lipooligosaccharides

The outer envelope of the *Brachyspira* species is considered to contain lipoologosaccharide (LOS) rather than lipopolysaccharide, based on the lack of a typical Gram-negative ladder formation of repeated O-sugar components on silver-stained SDS-PAGE gels [54], [55]. LOS is considered to be involved in the induction of colonic lesions associated with *B. hyodysenteriae* [56], [57], and hence should be considered as potentially being involved in virulence in other *Brachyspira* species. LOS is also antigenic, and protective immunity to *B. hyodysenteriae* is specific to the LOS serogroup [58]. The importance of LOS in relation to protective immunity to *B. pilosicoli* is less clear, as pigs experimentally infected with *B. pilosicoli* 95/1000 did not develop a systemic antibody response against the spirochete [59].

**Figure 9:**
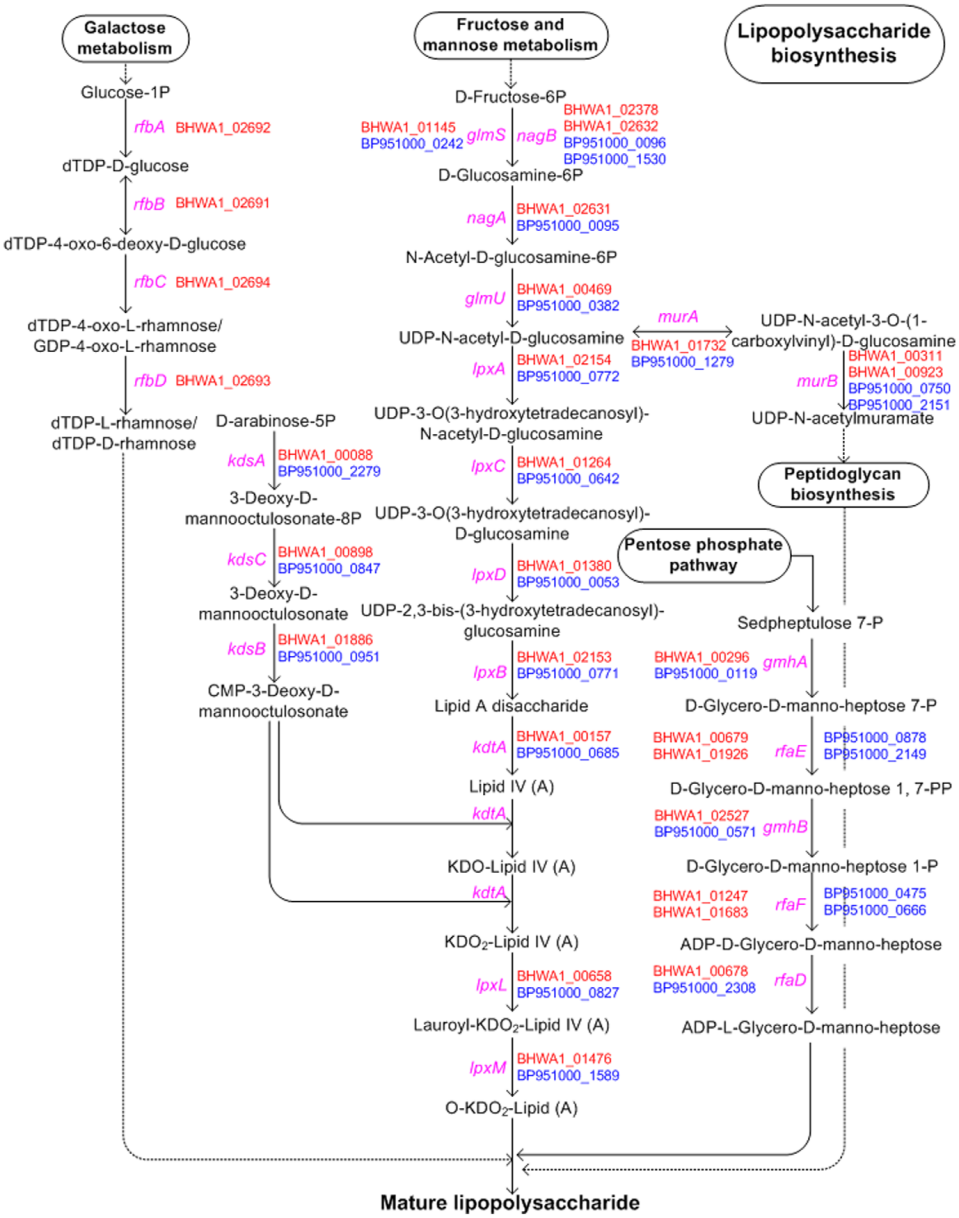
Different biosynthetic pathways for lipooligosaccharide in *B. pilosicoli* 95/1000 and *B. hyodysenteriae* WA1. The gene name is shown in pink and corresponding predicted ORFs present in the *B. hyodysenteriae* genome are indicated in red, and those for *B. pilosicoli* in blue.

*B. pilosicoli* contained a set of 25 core genes involved in the biosynthesis of LOS (Table 3). This was fewer than the 30 genes identified for *B. hyodysenteriae*, but more than the 10 so far identified in *B. murdochii*. The predicted pathways for biosynthesis of LOS and peptidoglycan for *B. hyodysenteriae* and *B. pilosicoli* are shown in Figure 9. The main difference between the species related to the presence of an *rfbBADC* cluster of genes on the ∼36 kB plasmid in *B. hyodysenteriae* WA1 that encode proteins for nucleotide sugar biosynthesis (dTDP-rhamnose) that are likely to be involved in O-antigen assimilation. A similar *rfbBADC* cluster was not found in *B. pilosicoli* or in *B. murdochii*, implying that they were unable to produce these antigens. Similar *rfb* gene clusters have been linked to virulence in numerous Gram negative bacteria [60], [61], and, for example, are plasmid encoded in the case of some *Salmonella enterica* serovars [62]. Further work is required to examine the potential role of the *rfb* cluster in the virulence of *B. hyodysenteriae*. It was interesting that despite the presence of genes encoding glycosyltransferases in this pathway, *B. hyodysenteriae* does not appear to produce ladder-like O-antigens [54]. This discrepancy requires further investigation. The most precise way to describe the diversity of LPS/LOS in the *Brachyspira* species will be to make a detailed comparison of polysaccharide content and structures. It is unfortunate that no such structural data currently are available.

#### Chemotaxis

The capacity for chemotactic responses and motility are extremely important for *Brachyspira* species, allowing them to colonize specialized niches in the complex nutritional, physical and microbiological environment of the large intestine. Interestingly, a striking difference was found in the numbers of chemotaxis genes. *B. hyodysenteriae* contained 52 genes associated with chemotaxis, compared to 27 in *B. pilosicoli* and 39 in the partial genome of *B. murdochii* [Table 3]. The distribution of methyl-accepting chemotaxis genes also was different between the species, with *B. pilosicoli* having no *mcpC* genes. These differences are likely to be highly significant in relation to the chemotactic signals that the different species can respond to: they may explain the differences that have been observed in the attraction of *B. pilosicoli* and *B. hyodysenteriae* to mucin, and their tendency to occupy different local niches within the large intestine [63]. *B. hyodysenteriae* colonizes the lumen of the colon, but also penetrates deep into the colonic crypts, where it enters goblet cells. Although *B. pilosicoli* also can enter the crypts, unlike *B. hyodysenteriae* it penetrates the dense mucus layer at the crypt shoulders, and attaches to the underlying colonic enterocytes. As *B. hyodysenteriae* has a much more limited host range than *B. pilosicoli*, it seems paradoxical that it has a greater number and variety of methyl-accepting chemotaxis genes.

*B. pilosicoli* contained 13 chemosensory transducer genes, and it was interesting that seven of these, and genes for five hypothetical proteins, were located within a cluster at a ∼5.7 Kb locus. This locus comprised *cheB* (BP951000_0460), *cheD* (BP951000_0461), *cheR* (BP951000_0462), *cheW* (BP951000_0463), *cheB* (BP951000_0464), *cheY* (BP951000_0465, five copies of hypothetical proteins (BP951000_0466 to BP951000_0470), and *cheX* (BP951000_0471). Similarly, six of the chemosensory transducer genes of *B. hyodysenteriae* were within one cluster at a locus of similar size. These genes encoded chemotaxis response regulator (*cheY*, BHWA1_00488), chemotaxis histidine kinase (*cheA*, BHWA1_00489), chemotaxis protein (*cheW*, BHWA1_00490), chemotaxis protein methyltransferase (*cheR*, BHWA1_00491), chemotaxis protein (*cheD*, BHWA1_00492), and response regulator receiver modulated CheB methylesterase (*cheB*, BHWA1_00493). The other chemosensory transduction genes were located elsewhere on the two genomes, and a similar clustering of *che* genes was not found in *B. murdochii*.

#### Motility

Only minor difference were found in the number and types of flagellar-associated genes between the three *Brachyspira* strains (Table 3). *B. hyodysenteriae* WA1 and *B. murdochii* 56-150^T^ contained four genes for FlaB core proteins while *B. pilosicoli* 95/1000 contained three. WA1 and 95/1000 contained three FlaA sheath protein genes while 50-150^T^ contained two (Table 3). 50-150^T^ also contained two copies of fleN, encoding flagellar synthesis regulator FleN, while the other two strains had single copies. As expected, the three strains had many minor differences in the predicted sequences of the flagellar proteins. When considering the predicted flagellar structure, it should be remembered that *B. pilosicoli* only has 4–6 periplasmic flagella at each cell end compared to 7–14 for *B. hyodysenteriae* [1], and this difference could involve the need for some modifications. Furthermore, differences in flagellin protein ratios can affect the stiffness of the periplasmic flagella in spirochetes, and this stiffness directly correlates with their motility [64]. Hence, depending on expression of the flagellin proteins, the species are predicted to have different capacities for motility.

#### Lipoproteins and surface proteins

As with other bacteria, predicted surface associated proteins and lipoproteins of the *Brachyspira* species are likely to be important in interactions with the host; for example, cell surface proteins are thought to be involved with the polar attachment of *B. pilosicoli* to colonic enterocytes [65]. *B. pilosicoli* contained substantially fewer genes encoding lipoproteins than did *B. hyodysenteriae* (13 versus 34). Such lipoproteins, where they are surface associated, are likely to be important as potentially targets for immune recognition and could be used as subunit vaccine candidates [66]. An example of the difference between the two fully sequenced *Brachyspira* species was the presence of a gene encoding a peptidoglycan-associated outer membrane lipoprotein (PAL, BHWA1_02111) in *B. hyodysenteriae* that was absent in *B. pilosicoli*. PAL is involved in maintenance of the integrity of the cell envelope [67], and contributes to bacterial virulence and inflammation in Gram-negative sepsis [68]. Examples of lipoprotein-encoding genes that were shared between the species include those for basic membrane lipoprotein, outer membrane lipoprotein, apolipoprotein N acyltransferase, lipoprotein releasing system, transmembrane protein, LolC/E family, and lipoprotein releasing system ATP binding protein. The species had other predicted lipoproteins, but these were of unknown function.

*B. hyodysenteriae* has been reported to have a set of variable surface proteins of about 39 KDa [69], and strain WA1 contained nine genes encoding these proteins (VspA to VspF, and VspH), with two copies of VspA and VspD. Genes for VspA to VspF were located adjacent to each other (BHWA1_01456 to BHWA1_01601), whilst those for one copy of VspA and for VspH were located elsewhere on the genome (BHWA1_00889 and BHWA1_02382, respectively). *B. pilosicoli* contained single copies of the genes for VspD, VspE and VspH located in different regions, while *B. murdochii* 56-150^T^ had three copies of *vspG*, two of *vspF* and one of *vspG*. Differential expression of these genes may be involved in immune evasion, allowing persistence of the spirochetes [69].

Amongst the other outer membrane proteins, *B. pilosicoli* 95/1000 contained three copies of *bspA*, predicted to encode a BspA-like surface protein. BspA-like proteins are expressed on the surface of many pathogenic bacteria: they may bind to fibronectin, stimulate a significant serological response [70], and induce secretion of IL-8 [71].

#### Hemolysis-associated genes

*B. hyodysenteriae* is strongly hemolytic, whereas *B. pilosicoli* and *B. murdochii* are weakly hemolytic [1]. The strong hemolytic activity of *B. hyodysenteriae* is thought to be an important virulence trait [72], [73]. In addition to the seven genes associated with hemolysis previously identified in the genome of *B. hyodysenteriae* WA1 [15], an additional putative hemolysin gene was identified (BHWA1_00962).

Only single copies of *tlyA*, *tlyB*, *tlyC*, and *acpP* (also known as *hlyA* [73]) were identified in the partial genome of *B. murdochii*. On the other hand, *B. pilosicoli* 95/1000 contained eight genes potentially involved with hemolysis, of which seven were similar to those in *B. hyodysenteriae*. These encoded hemolysin A (*tlyA*, BP951000_0123), ATP-dependent Clp protease proteolytic subunit (*clpP*, also known as *tlyB*, BP951000_1802), hemolysin C (*tlyC*, BP951000_1288), hemolysin related protein (*hly*, BP951000_0473), acyl carrier protein: contains beta-hemolysin (*acpP* or *hylA*, BP951000_0533), putative hemolysin III (BP951000_0424), and putative channel protein hemolysin III family protein (BP951000_0888). The eighth was a putative hemolysin (BP951000_2207) that was similar to a protein of *Parabacteroides distasonis* ATCC 8503, with ∼42.9% protein identity. *B. pilosicoli* lacked a gene with similarity to that encoding the new putative hemolysin of *B. hyodysenteriae* (BHWA1_00962). From this, it is possible that the new putative hemolysin identified in *B. hyodysenteriae* is responsible for its strongly hemolytic phenotype. Alternatively, it has been suggested that differences between *B. hyodysenteriae* and the weakly hemolytic *Brachyspira* species such as *B. pilosicoli* in the FabF ACP synthase and FabG ACP reductase proteins that flank the acyl carrier protein (HlyA) may result in different lipid moieties being attached to the HlyA proteins [32]. Acyl carrier protein-dependant fatty acylation is known to be important in activation of prohemolysin to active hemolysin in *E. coli* [74], and such differences therefore may be important for expression of the strongly hemolytic phenotype in *B. hyodysenteriae*.

#### Proteases

*B. hyodysenteriae* WA1, *B. pilosicoli* 95/1000 and *B. murdochii* 56-150^T^ contained similar numbers of genes predicted to encode proteases (15, 15 and 14, respectively). The proteolytic capacity in all three species was linked with the large number of ORFs encoding enzymes involved in uptake and metabolism of amino acids. These proteases are likely to be required for survival in the intestinal environment, but also may be involved in induction of local destruction of host tissues, and hence contribute to virulence. A serine protease previously described in *B. pilosicoli* (BP951000_0826, BP951000_1141 and BP951000_2083) [75] was found to have >82% sequence identity to proteases of *B. murdochii* 56-150^T^ (ZP_04048074) and *B. hyodysenteriae* WA1 (YP_002721653).

#### Secretion systems

As with *B. hyodysenteriae* [15], *B. pilosicoli* lacked genes for the specialized secretion systems found in pathogenic Gram-negative bacteria, although it had genes for the Sec pathway and ABC-type transporters. Six *sec* genes were identified: *secA* (BP951000_0230), *secY* (BP951000_1006), *secF* (BP951000_1532), *secD* (BP951000_1533), *secE* (BP951000_1559) and *secG* (BP951000_1896), and there were 36 genes encoding ABC transporters (compared to 35 and 29 for *B. hyodysenteriae* and *B. murdochii*, respectively). Ten flagella-associated genes that can form part of a type III secretary system were found in *B. pilosicoli* 95/1000, but needle associated genes that encode proteins required for the injection of toxins into the host cell were not identified.

### Central metabolic pathways

**Figure 10:**
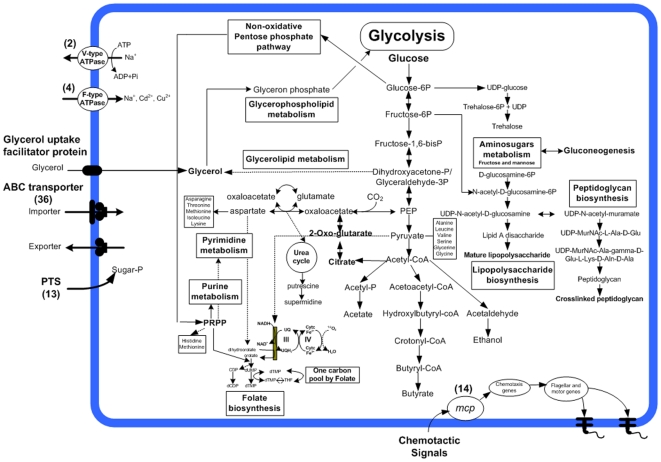
Central metabolic pathway construction for *B. pilosicoli* 95/1000.

A reconstruction of the central metabolic pathways of *B. pilosicoli* showed that it shared many metabolic capabilities with *B. hyodysenteriae*. The pathways for *B. pilosicoli* are shown in Figure 10, and can be compared with those for *B. hyodysenteriae* by examining Figure 2 in reference 15. In both species the glycolysis-gluconeogenesis metabolic axis constituted the backbone of energy production and the starting point of many biosynthetic pathways. The biosynthesis of peptidoglycan, phospholipids, aromatic amino acids, fatty acids and cofactors commenced from pyruvate, or from intermediates in the glycolytic pathway. As with *B. hyodysenteriae*, a complete set of genes for the non-oxidative pentose phosphate pathway, nucleotide metabolism and a respiratory electron transport chain were identified in *B. pilosicoli* and *B. murdochii*. It can be seen from the figures that some of the obvious ways in which *B. pilosicoli* differed from *B. hyodysenteriae* related to the presence of pathways associated with citrate and 2-oxo-glutarate, a pathway from acetyl-CoA to ethanol, and a lack of the *rfbBADC* genes located on the *B. hyodysenteriae* plasmid (predicted to be involved in O-antigen biosynthesis).

Unlike *B. hyodysenteriae*, *B. pilosicoli* and *B. murdochii* both appeared to have the capacity to use enzymes of the incomplete TCA to synthesize glutamate. Relevant predicted enzymes in the two species were putative citrate synthase (*gltA*, BP951000_0367 and 4083292.C42.orf00523), putative isocitrate dehydrogenase (*idh*, BP951000_0368 and 4083292.C42.orf00525), aconitase (BP951000_0369 and 4083292.C42.orf00530), malate dehydrogenase (*mdh*, BP951000_1737 and 4083292.C15.orf00095), and two copies of fumarate hydratase in *B. pilosicoli* (*fumA*, BP951000_1272 and BP951000_1273): *fumA* was not indentified in *B. murdochii*, possibly due to the genome being incomplete. This capacity emphasized the dual function of the TCA portion of the cycle: provision of NADH as part of the complete cycle in aerobes, and provision of alpha-oxaglutarate in aerobes and anaerobes. Aconitase allows the TCA cycle to function during periods of oxidative stress, and may play a role in mediating oxidative stress and regulating cell death [53], [76]. The presence of malate dehydrogenase as an alternative enzyme for converting oxaloacetate into malate is likely to provide a source of malate for cell biosynthesis in *B. pilosicoli* and *B. murdochii*.

A key branch in the partial TCA cycle of *B. pilosicoli* and *B. murdochii* involved 2-oxoglutarate, succinyl-CoA and fumarate, precursors that could proceed in either the oxidative or reductive direction. Unlike *B. hyodysenteriae*, *B. pilosicoli* and *B. murdochii* also had a gene set for the production of ethanol from acetyl-CoA via a branched fermentation pathway. The significance of this difference between the species is unknown, but it is likely to influence overall metabolic flexibility.

*B. pilosicoli* can be distinguished from all the other *Brachyspira* species in that it lacks beta-glucosidase activity [77]. Consistent with this, no genes specifically encoding beta-glucosidase were identified in the *B. pilosicoli* genome; however, a novel system for metabolizing multiple sugars, including beta-glucosides was identified that involved alpha-galactosidase/6-phospho-beta-glucosidase (*celF*, BP951000_0253), PTS system, arbutin-like IIC component (*glvC*, BP951000_0254) and 6-phospho-alpha-glucosidase (*bglA*, BP951000_0255). These enzymes have specificity for 6-phospho-beta-D-glycoside substrates rather than the beta-D-glycoside substrates of beta-glucosidases [78], [79]. Genes associated with beta-glucoside metabolism are likely to be important in *Brachyspira* species as they are known to regulate growth, adhesion and colonization in other bacterial species [80].

*B. hyodysenteriae* and *B. murdochii* had genes encoding phosphoenolpyruvate synthase, whereas *B. pilosicoli* did not appear to have the capacity to interconvert phosphoenolpyruvate (PEP) and pyruvate. Instead, it may utilize glycerate but not pyruvate for growth, and use pyruvate kinase (*pyk*, BP951000_0535) to catalyse the reverse conversion of PEP to pyruvic acid [81]. This process increases markedly under microaerobic conditions, with a corresponding increase in the rate of constructive metabolism.

*B. hyodysenteriae* and *B. murdochii* possessed a complete set of genes for histidine metabolism, while *B. pilosicoli* did not. It is possible that *B. pilosicoli* has an alternative pathway for synthesizing histidine. It may however also obtain nutrients from the surrounding environment, particularly as the intestine is a rich source of amino acids, including histidine [82]. Consistent with this requirement, three copies of a polar amino acid uptake ABC transporter, PAAT family, amino acid-binding protein (BP951000_0988, BP951000_0989 and BP951000_0990) were identified in *B. pilosicoli*, and not in the other two species.

A complete fatty acid biosynthesis gene set previously has been found in *B. hyodysenteriae* [15], and was identified here in *B. murdochii*. In contrast, an incomplete gene set was identified in *B. pilosicoli*, with only the genes for (3R)-hydroxymyristoyl-(acyl carrier protein) dehydratase (*fabZ*, BP951000_0773), malonyl CoA acyl carrier protein transacylase (*fabD*, BP951000_1348), and the previously mentioned locus comprising ACP synthase II (*fabF*, BP951000_0532), acyl carrier protein (*acpP*, BP951000_0533) and acyl reductase (*fabG*, BP951000_0534) being identified while FabZ provides the 3-hydroxymyristate moieties characteristic of lipid A. FabD is involved in elongation reactions in fatty acid synthesis. These findings suggested that *B. pilosicoli* does not incorporate carbon from simple carbon sources into fatty acids.

#### Implications

In this study the complete genome sequence of *B. pilosicoli*, a widespread but somewhat neglected enteric pathogen of humans and animals, was obtained and made available for analysis by the scientific community. The spirochete genome was shown to have basic similarities to the genomes of the related species *B. hyodysenteriae* and *B. murdochii*, but a number of distinct features were identified that may help to explain the different host ranges and colonization sites of the species. These included predicted differences in chemotactic responses, oxygen sensitivity, the use of some different substrates, the presence of different surface proteins, and predicted differences in LOS content and structure. The first description of genes for an apparently complete bacteriophage in *B. pilosicoli* also was made: this finding has important practical implications, since if the bacteriophage can be shown to be functional it could be used for genetic manipulation of *B. pilosicoli*. The current lack of such a genetic tool for *B. pilosicoli* is a constraint that limits functional studies of the genes that have been identified.

## Supporting Information

Figure S1 Whole-genome comparisons between *B. pilosicoli* 95/1000 and *B. hyodysenteriae* WA1 displayed using the Artemis Comparison Tool. Regions of DNA:DNA similarity are joined by lines.
(2.24 MB TIF)
