# Supplementary figures and images for: The Complete Genome Sequence of the Pathogenic Intestinal Spirochete Brachyspira pilosicoli and Comparison with Other Brachyspira Genomes
Source: PLoS One. 2010 Jul 6;5(7):e11455. doi: 10.1371/journal.pone.0011455 (PMC2897892; doi:10.1371/journal.pone.0011455)

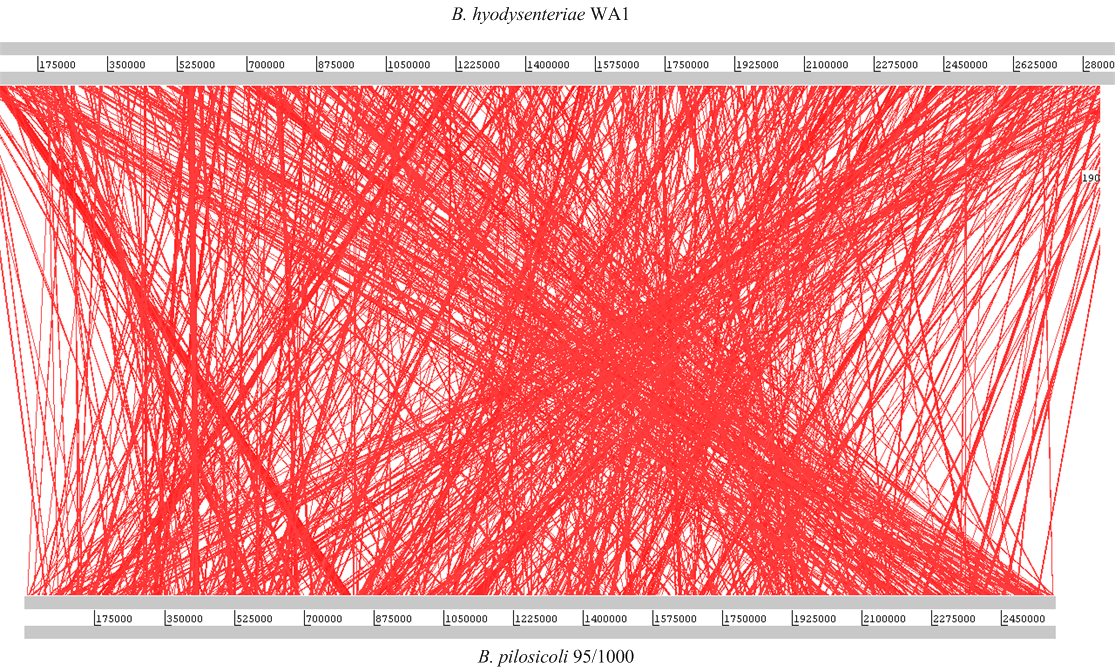

Supplement: Figure S1 — Whole-genome comparisons between B. pilosicoli 95/1000 and B. hyodysenteriae WA1 displayed using the Artemis Comparison Tool. Regions of DNA:DNA similarity are joined by lines. (2.24 MB TIF) [file pone.0011455.s001.tif]
